# Perceptions of medical students and their mentors in a specialised programme designed to provide insight into non-traditional career paths

**DOI:** 10.5116/ijme.4e27.00bb

**Published:** 2011-07-24

**Authors:** Hedieh Asadi, Anna Josephson, Terese Stenfors-Hayes

**Affiliations:** Department of Learning, Informatics, Management and Ethics, Karolinska Institutet, Sweden

**Keywords:** Mentor, medical student, career path, mentor programme

## Abstract

**Objectives:**

This pilot study explores the perceptions of medical students and their individual mentors who advised them in a specialised programme where students gained insight into non-tradition career paths.

**Methods:**

Twelve medical students in years 3-6 at Karolinska Institutet, Sweden were recruited to the Prominentia mentor programme where they were individually paired with mentors who met with them to discuss and advise them on non-traditional career paths. Application letters of students to join the programme as well as electronically distributed questionnaires and semi-structured interviews were used to assess the perceptions of mentors and students to the programme. Both the questionnaire and the interview transcripts were thematised using content analysis.

**Results:**

In terms of expectations and requests, the application letters showed that all students specified their career goals and the type of mentor they desired. Whereas mentors in general had fewer requests and some had no specific demands. In light of perceived effects, all mentors felt they discussed future careers with their students and the majority of students responded the same way, with some interesting deviations. Most discussed topics during meetings were: future career, medical education, combinations of private life and work, and work environment.

**Conclusions:**

This pilot study revealed that students appreciated receiving inspiration and seeing career path opportunities outside academic medicine as well as receiving support in personal and professional development and guidance about the students’ role as a doctor. However, discrepancies were found regarding how mentors and students respectively perceived the mentor programme.

## Introduction

For several decades, it has been acknowledged that mentor programmes can produce many positive outcomes.^1^ Traditionally, mentors help students to develop their career goals and their ability to navigate psychosocial issues such as those surrounding gender and professionalism.^2^ Mentor programmes for doctors and undergraduate medical students are becoming increasingly available 3,4 and mentoring in undergraduate medicine is deemed important for career guidance, as well as personal development.^5^-^7^ However, academic institutions often undervalue mentorship, leading to a deficit in formal mentor programmes at most universities.^7^,^8^ Furthermore, mentor programmes for medical students usually focus on the typical career path such as going from a medical student to an intern, resident, and finally a specialist.A Swedish survey showed that almost 90% of residents have considered an alternative career outside the clinical field, in the private sector, or abroad.^9^ However, mentor programmes that focus on possible career paths outside the clinical field appear to be rare^7^ and students often find it difficult to find mentors who will help them pursue careers outside academic medicine.^9^,^10^ This indicates that there is a need for specialised mentor programmes that focus on non-traditional careers for medical school graduates. To meet this need, a specialised mentor programme named Prominentia was founded in Stockholm, Sweden at Karolinska Institutet during 2008 by a medical student. The mentor programme aims to support medical students who are interested in non-traditional medical careers. This study evaluates the first pilot year of this specialised mentor programme from the perspective of both the mentors and the participating students. Innovations in medical education today are commonly only explored from either the teachers’ or the students’ perspective using a qualitative or a quantitative approach. This study aims to evaluate the mentor programme and explores if a mixed methods evaluation using respondents from both perspectives (mentors and students) can provide additional insights. By using the same instrument as in previous mentor evaluations, this specialised mentor programme can also be compared to outcomes of previous, more general programmes.

## Methods

### The Prominentia mentor programme

Clinical rotations at Karolinska Institutet begin in third year of medical school and this experience often helps students decide a career path. Therefore medical students in their third through sixth year (approximately 1000 students) were targeted for the programme. They received an e-mail inviting them to apply for a total of 12 mentee positions within the mentor programme. The e-mail described the programme as an extracurricular activity and listed the aims of the programme. All the mentors had a medical (MD/PhD) background and leading positions in management consulting, venture capital, pharmaceutical/biotechnology companies, or the public health sector. The mentors worked at one of four companies (the Boston Consulting Group, Healthcap, Roche, and AstraZeneca) or at Karolinska Institutet. The project manager of the mentor programme selected the mentors from Karolinska Institutet and each company provided two mentors.The project manager of the mentor programme matched mentors and students using information gathered from the prospective mentors’ and students’ CVs and application letters. The students’ application letters included their career ambitions and explained why they wanted to participate in the specialised mentor programme, i.e., what they hoped to gain from having a mentor and how a mentor could assist them with specific needs related to over-all career goals. A total of 12 mentors were matched individually with 12 students. Three of 12 mentors and 8 of 12 students were female. Most students were in their mid-20s and the age of the mentors varied from 45- to 60-years-old. The programme lasted for one year, after which new mentees and mentors were recruited. The mentor and the student decided how often and how long they were to meet, but it was recommended that they meet once a month for two hours.A mentor consultant was hired to lead the mentor training for both mentors and students, which consisted of three-hour long sessions. The first session was an introduction to mentorship and included only the students. During this meeting, the students were given a chance to clearly define expectations and talk about any concerns they had related to the programme. In addition, the students introduced themselves to one another and discussed why they chose to participate in the programme. At the end of this meeting, the students received information about their mentor and were instructed to contact them. The second training session initially included only the mentors. During this part of the training session, the mentors were given a short introduction to mentorship and an opportunity to exchange experiences. After this introduction and exchange, the students joined the session. Next, both the students and the mentors shared why they chose to participate in the programme. By the time of the third training session, all mentors had met their mentees/students several times and the programme was evaluated and discussed by all participants. The three sessions were hosted by different companies that supported the programme. As an initial opportunity to expose students to different career options, each company gave a short presentation about their business and, in particular, how a medical degree could help someone contribute to their company.

### Study instruments

To evaluate the effects of the specialised mentor programme, three data sources were used: 1) application letters from both mentors and students, 2) questionnaire data from both mentors and students, and 3) interviews with five students/mentees. Three out of five interviewees and 7 out of 11 questionnaire respondents were female. Qualitative data from letters, questionnaires and interviews were analysed using content analysis.^11^ Ethical approval for this study was not required as recommendations for this type of study were followed according to current Swedish regulations.

The application letters were read by all three co-authors in an attempt to find differences and similarities in mentors and students expectations and requests regarding the programme. The questionnaire was developed in a previous research project.^5^ The development of the questionnaire was based on focus group interviews with mentors from two undergraduate mentor programmes and a literature review. The review showed that no previous questionnaire existed and hence this new instrument was developed. This instrument, which has been successfully used in two previous research projects^5^,^11^ was modified to fit the current specialised mentor programme. Two versions, one for mentors and one for students, were developed. The self-administered questionnaires included an e-mail explaining how the data would be used for evaluation, publication, and development of the programme. The questionnaires were distributed electronically (by one of the participating mentors) to all participants to respond to anonymously online. Three reminders were given and the questionnaire was open for four weeks. Eleven students and 9 mentors completed the questionnaire which included a mix of open-ended and close-ended questions. Some of the close-ended questions such as “what did you discuss with your mentor/student?” were based on a nominal scale and used qualitative variables. The response alternatives included future career, the medical education, and being a doctor. Other close-ended questions, e.g., “has the mentor given you guidance on your future career path?” were answered by the use of a Likert scale. These questions had response categories such as “not at all”, “to some extent”, “to a high extent”, and “to a very high extent”.The open-ended questions were analysed using content analysis^5^,^11^ by all three authors. The analysis started by reading through the material and identifying themes, similarities and differences in the answers both between mentors and students respectively and between the answers in the two separate groups of respondents. The three authors discussed the analysis until a negotiated consensus was reached.^12^In addition to the questionnaires, 5 of the 12 students were randomly selected and interviewed. The interviews were semi-structured and functioned as a follow-up to the questionnaire. In the interviews, questions such as “what was it like to have a mentor?” and “what did the mentorship mean to you?” were asked in order to obtain a better understanding of the answers on the original questionnaires. The first author (HA) performed the interviews. The interviews were recorded, transcribed, and analysed using content analysis by HA and TSH.^11^ The same method of analysis was used, by the three authors, for the open ended answers in the questionnaires. After identifying three main themes: expectation, the programme, and effects of the programme, subthemes were identified, compared, and described. Again, the authors discussed the analysis until they reached an agreement.^13^

## Results

A total of 24 people participated in the mentor programme. One mentor-student couple decided to end their participation in the programme, but were invited to take part in the study.

### Expectations and request

Analysis of the students’ application letters showed that all the students clearly specified their career goals and the type of mentor they desired (i.e., the type of experience their prospective mentor should have). Three out of 8 female students also wanted their mentor to be a woman with a leading position. According to the application letters, 5 students described the possibility to discuss future careers as the main reason for participating in the programme. For the other 6 students, being provided other options available with a medical degree was of greater importance. One student summed up her main objective this way:

“I would like to meet a mentor that does not necessarily open doors for me, but rather, shows me where the doors are and teaches me how to open them”.

The mentors had fewer requests regarding the type of student they wanted to meet. Three mentors did not have any specific demands; 4 mentors did not even answer the question. However, 5 of them identified specific desirable student characteristics: “positive attitude”, “innovative”, “sees opportunities and not obstacles”, and “interested in research”.When the students answered questions about why they wanted to be part of the mentor programme, 7 students responded with words related to widening their perspective and learning about alternative career paths. Two students mentioned reflection, discussion, and guidance as reasons for joining the programme. One student wanted to participate in the programme for personal development reasons and another student wanted to participate because the programme seemed interesting.

### Participating in the programme

The questionnaire data showed that most pairs met five to eight times. Four students answered that they would probably keep in touch with their mentor after the end of the programme. Three students were sure they would keep in touch, 3 were doubtful of future contact and one student did not think there would be any future contact. Seven of the students spent two to three hours (or more) each month on the mentor relationship. All responding students participated in the introduction training and the follow-up training, although only 7 students attended the last training meeting. According to the questionnaire findings, all but 2 of the responding students thought that the education/training for the mentors was sufficient. All of the responding students thought that the education provided for them was satisfactory. Ten of the responding students did not identify any problems with the programme.To explore whether mentors and students had perceived the mentor meetings in a similar manner, both mentors and students were asked what they had discussed in the meetings. The mentors’ and the students’ answers were similar and the most commonly discussed topics were future career, medical education, the combination of private life and work, and the working environment. All mentors thought they had discussed future career with their students and 10 of the responding students expressed the same view (Table 1).

**Table 1:** Discussion topics (n=24)

Topic	Students (%)	Mentors (%)
Career	91	100
The undergraduate programme	82	67
Combining work and private life	64	78
Work environment	64	22
Being a student	36	56
Inter professional cooperation	18	56
Ethical issues	18	33
Equality	27	0

Both the mentors and students were asked to reflect about the mentors’ role in their own words. The analysis showed that students mostly described the role of the mentor as a sounding board. Support, encouragement, reflection, and appreciation of the mentor’s experiences were other common descriptors. Similarly, the mentors also used the metaphor of a sounding board to describe their role. The mentors also described their role as helping their student find their own solutions. To further explore the role of the mentor, the respondents were asked to describe the mentor’s role using the suggested descriptors in Table 2.

**Table 2:** The role of the mentor from students’ and mentors’ perspectives

What was the role of your mentor?(Student’s perspective)	%	What was your role as a mentor? (Mentors’ perspective)	%
Relate to my situation and offer perspectives	91	Relate to my situation and offer perspectives	89
Facilitate personal and professional development	76	Facilitate personal and professional development	89
Assess and give feedback	64	Assess and give feedback	44
Being a role model	46	Being a role model	56

When asked in the questionnaire to complete statements such as “I think it is (fill in appropriate word) to have a mentor”, words such as stimulating, privileged, and safe were the most common responses by the students. In the interviews, the students explained that they had interpreted the objectives of the programme as a chance to discover new perspectives and opportunities available with a medical degree. However, one student noted that she did not experience any pressure to reach an objective, but felt that she was expected to set her own goals and objectives. Another student expressed that he already had his individual objectives and did not recall the objectives of the programme.

### Perceived effects of having a mentor

When students were asked in the questionnaire to describe, using their own words, what the mentor programme meant for them, they expressed the value of seeing different career opportunities, meeting like-minded participants, networking, and becoming more certain about future choices. These answers were also given during the interviews where all students emphasised the value of receiving inspiration and seeing the different opportunities available. For some, the programme was an eye opener. However, satisfaction with the mentor serving as general support and as a concerned listener was seen as even more important:

*“[It] was very nice to meet and talk about issues that concerned yourself with someone that is wise and has a lot of experience. It is a great privilege to have time set aside to talk about your own thoughts about life; this is something you very seldom do.”* - Female student

*“During my medicine rotation, I started to think about what I really wanted. I did not feel at home at the medicine ward and it was not how I had imagined the profession of a physician. And I was thinking about whether I really wanted to become a doctor at all. Having a mentor was very nice because I realised that there were so many opportunities available with a medical degree. Also, it was very nice to just sit with no prejudice and discuss yourself, your thoughts and what opportunities are available. So for me it was a rescue: my interest in the medical education and the medical profession came back.”*- Female student

The interviewees also valued the training and the social activities with the other participants:

*“[To] see that you are not alone at medical school with having a lot of other thoughts and ambitions.”* - Male student

*“It was fun meeting the other medical students that participated because everybody was very driven and outgoing and there were a lot of thoughts and ideas. There are not a lot of people who think outside the box, so it was fun meeting them.”-* Male student

The mentors, using open-ended questions, on the other hand, almost exclusively thought the mentor programme provided the students with information about the challenges associated with careers outside typical medical careers. The following comments from the mentors illustrate these points:

*“The effect of the program is certainly very individual, depending on where they are in life. The programme has succeeded to open up new perspectives on opportunities and in the best case scenario it may also provide new ways to think.”* - Male mentor

*“I think that I, as a mentor, have given valuable information about alternative career paths for doctors. I have provided guidance and coaching in several difficult choices and opportunities in career paths that came up, I have given feedback on ideas and made the student think in new ways, and given input on tactical choices.”-* Male mentor

In addition, questionnaires showed that ten students expressed that the mentorship to some extent led to increased reflection regarding their own values. Seven of the responding students also thought the programme had facilitated their personal and professional development to a high extent/to a very high extent. The same number of students claimed to have received guidance regarding the role as a doctor to some extent. Similarly, 8 of 9 mentors thought they had stimulated personal development and all responding mentors thought they had guided the students regarding their role as doctors and future career choices.

## Discussion

The effectiveness of traditional mentor programmes in medical education to support, for example, personal and professional development has previously been shown.^3^,^4^,^6^,^7^,^14^ The mentor programme Prominentia was designed to fill a gap in the medical undergraduate programme regarding the students’ future career possibilities. The programme was initiated, designed and run by a medical student. The intention of this mentor programme is to match mentors with students that have interests outside of academic medicine, which otherwise has been difficult to accomplish.^10^ In the letters of application, this was emphasised by the students, but the need for more general guidance and support was also mentioned. The evaluation of the programme showed that receiving inspiration and seeing opportunities was appreciated by the students. Nevertheless, many highly valued the chance to talk to someone senior, to receive general support, and to reflect upon their own experiences. The other objectives of the programme – personal and professional development and guidance about the students’ role as a doctor – were reached to a satisfactory level. According to both the questionnaire and the interviews, the students valued not only the influence from the mentor but also meeting other students with similar ideas. In addition, general networking with the whole group of mentors was seen as important. This also means that some of the positive effects may be linked to the shared training with all mentors and students rather than the individual meetings with the mentor. The data gathered from this study does not make it possible to separate the effects of the two.When comparing the current findings with two previous studies in other undergraduate mentor programmes, using a similar questionnaire, some similarities and differences can be seen. The most common topics discussed according to the mentors in all three programmes^5^,^11^ were career, balance of work and private life, the undergraduate programme, being a student, and the professional role. According to the mentors, career issues were discussed with all students in the current programme. In the two previous studies, which explore more general mentor programmes, career issues was only discussed by 60% and 80% respectively.^5^,^11^ Furthermore, when observing what role the mentors thought they played, the identified themes such as being a sounding board, providing support, encouraging independent thinking, and encouraging independent decision-making were similar to previous findings.^5^,^11^ This shows that the programme was successful in focusing on career issues, but also that the difference between Prominentia and other general mentor programmes may not be as big as expected. When applying for the programme, the participants may have written their applications to fit the purpose of the programme rather than to identify their own needs and wishes. Ideally, alternative mentor programmes should therefore be available to meet other needs.Data for this study is based on self-reports, which is the most common method for this type of studies.^15^ Previous studies in this field however tend to focus on either mentor or students, which results in a rather unilateral perspective. Although this current study included all 24 participants and had an acceptable response rate, more studies are needed to evaluate this approach. The Prominentia mentor programme is now running for its third year, and follow up studies of this first pilot study are underway. The participating companies may have had an invested interest in the programme that could have affected the outcomes. However, as the students were unavailable for employment for three to five years, this risk was deemed rather small.

## Conclusion

The mentoring program has been found to be very useful and informative. Evaluation of the program revealed that students appreciated receiving inspiration and seeing opportunities. Also, during this program they had a chance to talk to seniors and received general support with regards to personal and professional development and guidance about the students’ role as a doctor. Compared to previous studies, career issues were discussed to a larger extent. The mentors in Prominentia filled a somewhat different role in their own eyes than the role the students identified. The mentors wanted to show opportunities outside the typical clinical career path, but the students also used them for two other purposes: reassurance and general guidance. The findings of this study highlights the importance of gathering data from both perspectives, whether it is mentor and students or for example teachers and students, when evaluating innovations as the difference in these perspectives would not otherwise have been identified. With reference to the application letters, it is suggested that instead of asking students to adjust their needs to fit with the programs’ requirements they should have been asked to propose their own needs and wishes and it is recommended that mentor programs are designed to meet a variation of needs.

## 

### Conflict of Interest

The authors declare that they have no conflict of interest.
